# Use of Methylphenidate Analogues as Cognitive Enhancers: The Prelude to Cosmetic Neurology and an Ethical Issue

**DOI:** 10.3389/fpsyt.2019.01006

**Published:** 2020-01-23

**Authors:** Simona Zaami, Adriano Tagliabracci, Paolo Berretta, Francesco Paolo Busardò, Enrico Marinelli

**Affiliations:** ^1^ Department of Anatomical, Histological, Forensic, and Orthopedic Sciences, Sapienza University, Rome, Italy; ^2^ Section of Legal Medicine, Department of Excellence-Biomedical Sciences and Public Health, Ancona, Italy; ^3^ National Centre on Addiction and Doping, Istituto Superiore di Sanità, Rome, Italy

**Keywords:** cognitive enhancers, mental health, alterations, medical ethics, cosmetic neurology

## Introduction

Drugs involved in the treatment of Alzheimer’s disease and other cognitive deficits such as attention deficit hyperactivity disorder (ADHD), strokes, schizophrenia, and aging are medically defined as cognitive enhancers (1). Amphetamines were the first drugs used to stimulate memory consolidation and improve concentration, and were followed by non-amphetaminic central nervous system (CNS) stimulants modafinil and armodafinil, which are largely prescribed for the treatment of narcolepsy and ADHD, although their mechanism of action is not entirely understood. Atomoxetine, a selective nor-adrenaline reuptake inhibitor, has been used in children with medication-resistant ADHD or undergoing side effects related to other drugs, while donepezil, a second-generation acetylcholinesterase inhibitor, has been employed for the treatment of mild-to-moderately-severe symptoms of Alzheimer-related dementia (2).

Nevertheless, methylphenidate is undoubtedly the most prescribed cognitive enhancer. It is also the most misused. Indeed, the non-medical use of methylphenidate and cognitive enhancers in an attempt to improve memory, increase mental concentration, control anxiety, and stimulate motivation and creativity is a rising worldwide phenomenon (2, 3). Due to methylphenidate being a prescription drug with medical restrictions in several countries, many illegal analogues have emerged on the internet and darknet drug markets during the last few years (3).

The misuse of cognitive enhancers in adults and young healthy individuals with the aim of increasing neurological functions is part of a phenomenon defined as “cosmetic neurology” (2). In addition, psychedelic microdosing using small doses of psychedelic substances, such as LSD and psilocybin is becoming more common (4). However, this cognitive enhancement comes with mental and ethical costs.

### Risks for Physical and Mental Health of Cognitive Enhancers

The physical and mental health risks associated with the use of methylphenidate analogues such as ethylphenidate, 3,4-dichloromethylphenidate, 3,4-dichloroethylphenidate, 4-fluoromethylphenidate, 4-fluoroethylphenidate, methylnaphthidate, ethylnaphthidate, isopropylphenidate, propylphenidate, 4-methylmethylphenidate, and N-benzylethylphenidate were recently reviewed and several severe intoxications and fatalities were reported (5). Moreover, the neurological and psychiatric consequences due to the misuse of methylphenidate analogues have been carefully evaluated. Psychiatric manifestations such as impulsive behavior, verbal, visual and memory impairment, gambling, compulsive shopping, and hypersexuality have been demonstrated, especially in younger users, due to excessive dopaminergic stimulation (2, 6). Furthermore, “psychostimulants” also alter the glutamatergic system, which can result in the impairment of behavioral flexibility and lead to the development and/or potentiation of addictive behaviors. Methylphenidate was proven to lower drug abuse liability in patients with ADHD. Still, it may also lead to similar behavioral rigidity and increase the risk for addictive or obsessive-compulsive behaviors, since the drug impacts glutamatergic signalling (3, 5).

Ethical issues raised by cognitive enhancement have been debated for over a decade (7): foremost experts have identified multiple ethical concerns including risks to mental and health safety. In 2015, the US Presidential Commission for the Study of Bioethical Issues (Bioethics Commission) released a report on the issue of cognitive enhancement, reporting findings, and establishing recommendations for the scientific community (8). A major issue is the current medical acceptance, or even endorsement, of interventions intended to restore or sustain “normality.” Both explicitly and implicitly, such a stance arguably adheres to the idea of a set of essential sociocultural requirements to function “normally”, considering abnormal or antisocial any deviation from established standards (3).

Remarks from the Australian Alcohol and Drug Foundation (9) have cast doubts on the actual cognitive benefits of most enhancers, indicating that scientific studies showed only little to no benefits for cognitive enhancement in healthy individuals, while the associated side effects do pose health risks (10). Furthermore, granted that the use of cognitive enhancers may somehow help in masking fatigue, boredom or procrastination, there is no evidence to suggest that they can actually make people smarter. Moreover, their effects are apparently temporary, lasting until their metabolization and elimination (11). Some of these drugs can cause dependence and have a wide range of side effects. They can be particularly harmful to young people as brains are not fully developed until the age 25.

### Medical Ethics

In 2014, the Italian Code of Medical Ethics included for the first time a new article defining human enhancement and related medical practices (12). The article was meant to reflect the relationship between this new field of medical practice and professional ethics. The article allowed for medical treatments going beyond conventional therapeutic goals, as long as several ethical and clinical criteria were met. Nonetheless, the problematic application of these criteria to human enhancement generated issues warranting an in-depth reflection.

## Discussion

The 2014 article of the Italian Code of Medical Ethics was replaced in 2017 by two new articles (76 and 76 –BIS), which are currently enforced (13). The new articles focus on medical enhancement and cosmetic medicine, stating that medical doctors being asked to provide or prescribe cognitive enhancers must always act in adherence to the highest standards of respect and protection for human dignity, human identity and integrity, and the inherent genetic traits in accordance with the principles of proportionality and precaution.

Nevertheless, it was shown that off-label use of cognitive enhancers can be efficacious in the treatment of postoperative cognitive dysfunction after major surgery in elderly patients, significantly decreasing the incidence of neurocognitive deficits (14). Other neuroscientists reported benefits for jobs requiring adaptive learning or attention shifting under pressure and recommend further research on the safety and efficacy of cognitive enhancers (15).

In the meantime, more reliable means to enhance cognitive functions should be promoted and prioritized: education, constant intellectual exercise and learning, a rewarding social life, interaction, a stimulating and healthy lifestyle. Such an approach is certainly more demanding and time-consuming than taking a supposedly “enhancing” drug, but it is much better for the individual in terms of personal identity development, creation of satisfactory interrelationships, self-esteem, and self-fulfilment.

## Author Contributions

SZ and FB provided initial idea and construct of the opinion. PB, AT, and EM co-authored and edited the manuscript.

## Conflict of Interest

The authors declare that the research was conducted in the absence of any commercial or financial relationships that could be construed as a potential conflict of interest.
